# *Trypanosoma cruzi trans*-Sialidase as a Potential Vaccine Target Against Chagas Disease

**DOI:** 10.3389/fcimb.2021.768450

**Published:** 2021-10-26

**Authors:** Kelli Monteiro da Costa, Leonardo Marques da Fonseca, Jhenifer Santos dos Reis, Marcos André Rodrigues da Costa Santos, José Osvaldo Previato, Lucia Mendonça-Previato, Leonardo Freire-de-Lima

**Affiliations:** Laboratório de Glicobiologia, Instituto de Biofísica Carlos Chagas Filho, Universidade Federal do Rio de Janeiro, Rio de Janeiro, Brazil

**Keywords:** *Trypanosoma cruzi*, Chagas disease, sialic acid, *trans*-sialidase, vaccine

## Abstract

Chagas’ disease is caused by the protozoan *Trypanosoma cruzi*, described in the early 20^th^ century by the Brazilian physician Dr. Carlos Chagas. There was a great amount of research devoted to diagnosis, treatment and prevention of the disease. One of the most important discoveries made since then, impacting the understanding of how the parasite interacts with the host’s immune system, was the description of *trans*-sialidase. It is an unique enzyme, capable of masking the parasite’s presence from the host, while at the same time dampening the activation of CD8+ T cells, the most important components of the immune response. Since the description of Chagas’ disease in 1909, extensive research has identified important events in the disease in order to understand the biochemical mechanism that modulates *T. cruzi*-host cell interactions and the ability of the parasite to ensure its survival. The importance of the *trans*-sialidase enzyme brought life to many studies for the design of diagnostic tests, drugs and vaccines. While many groups have been prolific, such efforts have encountered problems, among them: the fact that while *T. cruzi* have many genes that are unique to the parasite, it relies on multiple copies of them and the difficulty in providing epitopes that result in effective and robust immune responses. In this review, we aim to convey the importance of *trans*-sialidase as well as to provide a history, including the initial failures and the most promising successes in the chasing of a working vaccine for a disease that is endemic in many tropical countries, including Brazil.

## Introduction

Chagas disease is an anthropozoonosis caused by the flagellated protozoan *Trypanosoma cruzi*. American trypanosomiasis received this name in honor of Dr. Carlos Chagas, who described in his work, in 1909, the etiologic agent of the disease, the evolutionary cycle of the parasite as well as the vectors and clinical manifestations of the acute phase (Chagas, 1909). According to the World Health Organization, Chagas disease is classified as a neglected tropical disease with an estimated 8 million people infected worldwide and almost 100 million at risk of infection, the majority being found in Latin America.

The main form of transmission is vector, predominant in rural areas with rudimentary infrastructure, promoting an environment favorable to the reproduction of vectors as well as proximity to the wild cycle (Bern et al., 2019). Due to prevention and chemical control policies adopted since 1975 by most Latin American countries cases of domestic vector transmission have decreased substantially, reducing the occupation and prevalence of the main vectors in these areas (WHO, 2017). Nonetheless, Chagas’ disease is considered a reemerging infection due to alternative transmission routes of *T. cruzi*. One of them is the migration of asymptomatic infected individuals to non-endemic areas, allowing the transmission of the parasite *via* blood, organ or tissue donation, especially in countries that do not routinely search for *T. cruzi* in samples (Angheben et al., 2015). Another route occur through the consumption of food contaminated with both the feces of vectors and the secretion of infected mammals (Pereira et al., 2009). In these cases, individuals receive a high parasitic burden, which results in a more severe acute clinical manifestation with a high mortality rate (Yoshida et al., 2011).

In addition, the phenomenon of ecological succession allows new species to occupy niches previously occupied by removed ones, demonstrating that some species of triatomines are highly anthropophilic with a great capacity to adapt to new habitats. The increase of temperature has a direct effect on the development of vectors whose feed more frequently, putting more people at risk of infection (Short et al., 2017). This is a growing concerning in non-endemic areas as the southern United States of America, due to the increase in infestation of these insects in recent years. Even when serological testing for Chagas disease started in 2008 for blood donors in the state of Texas, seropositivity for *T. cruzi* was found in 1 in 6500 donors (Garcia et al., 2015b), with 5 of these cases being postulated as autochthonous transmission (Garcia et al., 2015a). Moreover, bedbugs (*Cimex lectularius*) may have an impact on the recent epidemiology of Chagas disease, since the species has been reported as a potential vector by sharing eating patterns similar and infecting mice both oral and vectorially (Salazar et al., 2015).

*T. cruzi* infection presents distinct two phases. The acute phase is characterized by high parasitemia, which can be asymptomatic, symptomatic and in rare cases fatal (Bern et al., 2019). Acute phase is recognized in only 2% of patients, since it is usually asymptomatic or displays non-specific symptoms (WHO, 2017). The absence of treatment results in the evolution to the chronic phase. In the indeterminate form, most individuals remain asymptomatic over the years, although they present positive serology for *T. cruzi*. However, about 30% of patients may progress to the determinate form of Chagas’ disease, with cardiac, digestive or mixed clinical manifestations (Bern, 2015). Currently, there is no vaccine to prevent Chagas disease and chemotherapy is the only alternative for curing infected individuals. The success of treatment depends on stage of the disease, age of the patient and biochemical characteristics of the parasite strain (WHO, 2017). Benznidazole is the drug of first choice with a cure rate of approximately 80% when administered in the acute phase. In the chronic phase, its effectiveness is reduced, as the real benefits of the treatment are not clear since the damage to affected organs is irreversible (Bern et al., 2019; Francisco et al., 2020).

Protective vaccines against several microorganisms have contributed to public health policies, allowing the effective control of diseases such as rabies, polio, diphtheria, measles, smallpox and tetanus. However, there is an urgency for the designer of vaccines against several other microorganisms, mainly parasites that cause serious human infections and a great public expense, such as Chagas disease, Leishmaniasis and Malaria. The production of a vaccine emerges as an economically advantageous alternative due to the reduction of expenses with patients, as well as an alternative to the reduction in the number of deaths. In recent years, several groups have focused on the development of vaccines for different immunodominant epitopes found in *T. cruzi*. Here, we will focus on advances in *trans*-sialidase (TS) protein-based vaccines. Basically, immunization against infectious agents can be divided into whole cell, toxoid, subunit and virus-vectored vaccines (Delany et al., 2014). In the second-last group are protein-based vaccines, which are purified from the entire pathogen or produced by recombinant genetic engineering (Lee et al., 2018). In recent years, recombinant protein technology has become efficient, allowing for cost-effective production in bacteria, yeast and other expression systems (Francis, 2018). This type of vaccine is considered safer even in immunosuppressed individuals due to the lack of an infectious agent. However, they usually have low immunogenicity, requiring booster doses and/or the use of adjuvants (Vetter et al., 2018).

## *Trans*-Sialidase and Its Effects on the Immune System

Out of all *T. cruzi* virulence factors, TS is likely the most important and by far the most interesting (Freire-De-Lima et al., 2015). It was already a known fact that *T. cruzi* would sport sialic acid (Sia) residues in the epimastigote’s surface membrane (Pereira et al., 1980; Schauer et al., 1983), despite being incapable of synthetizing Sia by itself. Our group first described it in 1985, when Previato et al. proposed that *T. cruzi* was capable of incorporating Sia residues in α-2,3 bonds to its own surface glycoproteins (Previato et al., 1985) thanks to TS activity in a mechanism that was later demonstrated both *in vitro* (Zingales et al., 1987; Schenkman et al., 1991) and *in vivo* (Previato et al., 1990).

The TS genes are part of the largest multigene superfamily in *T. cruzi*, composed of 1430 genes and 639 pseudogenes in the genome of the CL Brener strain (Herreros-Cabello et al., 2020). However, this number can vary considerably depending on the strain analysed (Berna et al., 2018; Callejas-Hernandez et al., 2018; Herreros-Cabello et al., 2020). Its members are currently divided into eight groups and all share the characteristic motif of the TS and TS-like superfamily: VTVxNVxLYNR (Freitas et al., 2011). Only proteins that clustered in group I present *trans*-sialidase and/or neuraminidase enzymatic activities and gave the superfamily its name. Most members from group I are formed by two main regions: N-terminal catalytic region and C-terminal one, containing repeating 12-amino acid sequences, known as Shed Acute Phase Antigen (SAPA), and GPI anchor (Pollevick et al., 1991). From its 19 members listed in the genome, the known members SAPA and TCNA are expressed in trypomastigotes and TS-epi, in epimastigotes (Freitas et al., 2011; Herreros-Cabello et al., 2020). The catalytic domain is rich in aromatic amino acids and a substitution mutation, with Tyrosine342 replaced by histidine being the most common, results in an inactive isoform of TS (iTS) (Cremona et al., 1995). iTS functions as a lectin capable of binding Sia residues (Todeschini et al., 2002a), and although it does not have catalytic properties, it must have a role during the cell invasion process (Freire-de-Lima et al., 2015). TS activity has been considered essential for parasite invasion and perpetuation in the infected host (Teixeira et al., 2015).

Regarding the other groups, group II comprises the genes of the known GP85 surface glycoproteins, such as ASP-1, ASP-2, TSA-1, Tc85, SA85, GP82, GP90, among others, which are expressed in trypomastigotes and associated with *T. cruzi* adhesion and invasion. Group III genes are also expressed in trypomastigotes and are able to inhibit the complement system, protecting *T. cruzi* from lysis. Known members include CRP, FL160, CEA and TESA. Group IV presents as a representative sequence TsTc13 and has no known function yet. Members of groups V to VIII have the gene sequence identified in the genome, but their function has not yet been described (Freitas et al., 2011; Callejas-Hernandez et al., 2018; Herreros-Cabello et al., 2020).

TS groups are defined by specific motifs, with group I being found in all *T. cruzi* strains and in different species of the genus (Herreros-Cabello et al., 2020). However, groups II and V are the most abundant in the genome of CL Brener (Freitas et al., 2011) and of other strains genome (Herreros-Cabello et al., 2020). The TS and TS-like superfamily have highly antigenic peptides, capable of eliciting a robust humoral response (Freitas et al., 2011) and are vaccine candidates against Chagas disease, such as TSA-1 (De La Cruz et al., 2019; Dumonteil et al., 2020), ASP-1, ASP-2 (Garg and Tarleton, 2002) and CRP (Sepulveda et al., 2000) and others. Nonetheless, only group I members that exhibit enzymatic activity and are referred to here as TS protein are the focus of this review.

**Figure 1:**
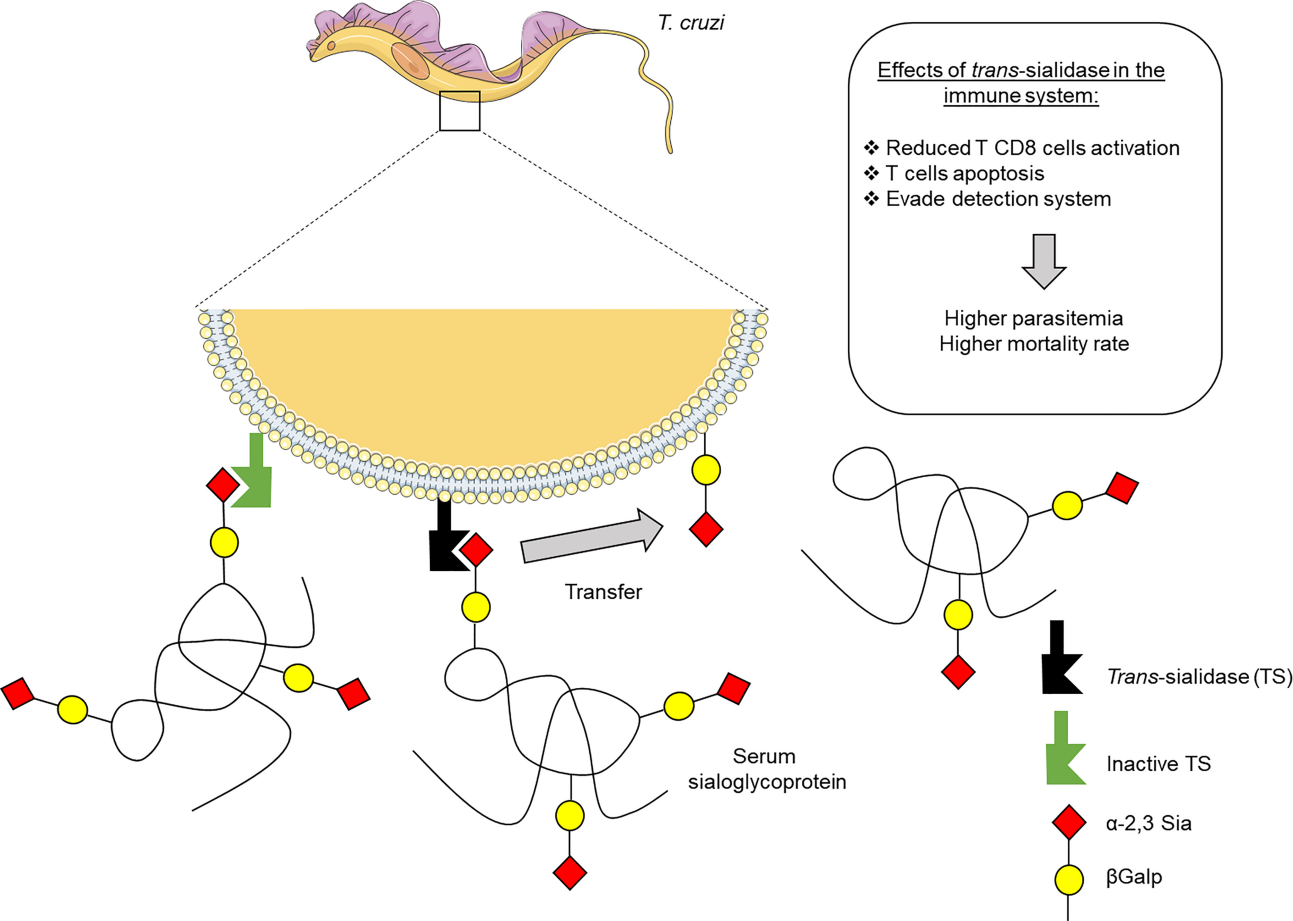
Effects of *Trypanosoma cruzi trans*-sialidase (TS) on the host immune system. The TS enzyme can be secreted by parasite or anchored in the membrane of trypomastigote forms of *T. cruzi*. The active form incorporates sialic acid from host sialoglycoproteins to acceptor molecules in the parasite membrane. The inactive form has no enzymatic activity and functions as a lectin. This sialylation allows the escape of parasite from detection by the immune system, allowing its survival and the establish the infection.

Throughout evolution, *T. cruzi* developed elegant mechanisms to disrupt the host immune response (**Figure 1**). Examples include its ability to induce anergy of T cells, as well as the production of low affinity antibodies (Oladiran and Belosevic, 2012; Nardy et al., 2016), which may be enabled by the action of TS proteins (Silva-Barrios et al., 2018; Da Fonseca et al., 2019). Since its discovery, several research groups have proposed molecular mechanisms displayed especially by the enzymatically active members to dampen the mammalian immune system, such as inducing apoptosis in thymocytes or even matures T lymphocytes (Mucci et al., 2002; Mucci et al., 2005) and also by dampening the ability of effector cells to combat the infection (Chuenkova and Pereira, 1995; Gao and Pereira, 2001; Gao et al., 2002; Freire-De-Lima et al., 2010; Bermejo et al., 2013; Nunes et al., 2013; Ruiz Diaz et al., 2015). In addition, given Sia’s ubiquitous distribution in the surface of mammalian cell, and its importance for both innate and adaptive immunity, it is not at all surprising to see that a foreign enzyme capable of such modulation of Sia expression has such ability to modulate the host’s immune system (Amon et al., 2014; Chen et al., 2014).

Our own group described how *T. cruzi*, due to TS activity is not only able to remove Sia from the host cells, but is also able to sialylate CD43 molecules present in the surface of T CD8 cytotoxic lymphocytes (Todeschini et al., 2002b; Mucci et al., 2006; Freire-De-Lima et al., 2010; Da Fonseca et al., 2019), this loss of Sia residues is one the necessary steps in the activation of these immune cells, since the presence of Sia prevents an effective interaction with MHC class I (Moody et al., 2001). Adding to that notion, the sialylation of CD43 in thymocytes by TS has been correlated to increased apoptosis in these immature cells, and not only that, but the use of TS inhibitors has been shown to halt the process (Mucci et al., 2006).

In a previous study, it was demonstrated that active TS induces the secretion of the cytokine IL-17 by B cells. Interestingly, such phenomenon was dependent on the sialylation of cell surface glycoconjugates, especially the CD45 glycoprotein (Bermejo et al., 2013). Although the mechanisms involved in many of those effects are not fully determined, it is clear they are dependent on the activity of *T. cruzi* TS, since they cannot be replicated by viral or bacterial neuraminidases and also, since TS does not affect mortality of SCID (severe combined immunodeficiency) mice, at least the main effects seem to involve mature host lymphocytes (Chuenkova and Pereiraperrin, 2005).

Given the many effects *T. cruzi* TS has on the host immune system and its importance to cell invasion, and adding the fact that there is no analogous enzyme in humans or other mammalian hosts, it does not come as a surprise that TS has always been considered a prime target for pharmacological intervention. Efforts to thwart TS effects in the immune system are not a recent novelty. Because sialoglycans play a crucial role in *T. cruzi* interaction and invasion events in host cells (Schenkman et al., 1993; Freire-De-Lima et al., 2017; Campetella et al., 2020), several groups, including ours, have searched for suitable strategies that could potentially be used to improve the outcome of Chagas patients. A previous study demonstrated that the use of neutralizing antibodies aimed at TS was effective in reducing parasitemia and mortality in mice, as well as preserved B cell areas both in ganglia and in spleen, and thymus architecture (Risso et al., 2007). Results published by our group confirmed the relevance of Sia on parasite-host cell contact from the use of a *Vibrio cholerae* neuraminidase suicide-type inhibitor (Carvalho et al., 2010). In this study, we demonstrated that besides inhibiting both TS and neuraminidase activities, the suicide-type inhibitor significantly reduced the parasite survival in *T. cruzi*-infected cells (Carvalho et al., 2010). In fact, over the last fifteen years, many natural and/or synthetic molecules have been tested as specific inhibitors (Buchini et al., 2008; Arioka et al., 2010; Carvalho et al., 2010; Giorgi et al., 2010; Lieke et al., 2011; Chen et al., 2018; Vazquez-Jimenez et al., 2021) of *T. cruzi*-TS activity, aiming towards the discovery of new agents for the cure of Chagas’ disease, but so far, a strong inhibitor has not been found. Since Sia cannot be synthesized by the parasite, and acts as an essential molecule for both communication and invasion of host cells, further studies are needed to better understand the catalytic mechanisms of the enzyme to favor the rational design of potential TS inhibitors.

## *Trans*-Sialidase Based Vaccine

In 1912, Blanchard demonstrated that animals surviving acute infection with *T. cruzi* were resistant to reinfection. Since then, active immunization against Chagas disease has been investigated besides methods to prepare the parasite for inoculation. Later, Pizzi and Prager used for the first time the cultivated and attenuated forms of *T. cruzi* to induce immunization and protect animals from infection by virulent strains (Rodriguez-Morales et al., 2015). Currently, there are several vaccine formulations against Chagas disease tested in animal model in the pre-clinical phase. Dumonteil and Herrera listed the recent formulations and platforms against *T. cruzi*, being 8 therapeutic and 19 protective vaccines (Dumonteil and Herrera, 2021). However, no vaccine is in clinical phase according to data from the *Clinicaltrials.gov* website. The main candidates for vaccines against infectious diseases already in clinical studies are based on attenuated microorganisms, DNA, viral vectors and recombinant proteins. Although there are many vaccines in phase one of the clinical trial, only vaccines with attenuated microorganisms and recombinant proteins are advancing to phases two and three. Possibly because DNA vaccines have shown low immunological efficacy in humans, although several strategies are being investigated to increase their immunogenicity (Suschak et al., 2017). Vector vaccines present greater safety issues due to immune cross-reactivity with the vectors and, consequently, the presence of adverse effects (Delany et al., 2014). As a result of the particularities of *T. cruzi* subculture that has an obligatory intracellular cycle, vaccines with live parasites pose a challenge for large-scale production as well as storage and distribution. Thus, vaccine candidates with recombinant proteins present a more promising proposal for success against *T. cruzi*.

In the 1990s, the development of DNA vaccines against infections caused by *Plasmodium yoelli* (Sedegah et al., 1994) and *Leishmania major* (Xu and Liew, 1995) showed great results of immunogenicity. In 1998, a study addressed the question of whether DNA vaccination could elicit immunization against experimental *T. cruzi* infection using as antigen the enzyme TS (Costa et al., 1998). Possibly, the idea of using TS came from several evidences suggesting that this enzyme is a virulence factor involved in the establishment of the infection (Chuenkova and Pereira, 1995; Leguizamon et al., 1999; Mucci et al., 2002; Tribulatti et al., 2005). Furthermore, a year earlier, Franchin and collaborators demonstrated that the passive transfer of monoclonal antibodies (MAb) recognizing sialic acid-dependent epitopes on the surface of bloodstream trypomastigotes was able to reduce parasitemia and the number of *T. cruzi* parasites found in the heart, skeletal muscle and liver, increasing the survival of immunized and challenged animals (Franchin et al., 1997). This reduction was specific since the passive transfer of MAbs recognizing the C-terminal repeated region of the TS did not produce the same effects. Thus, Costa et al. demonstrated that active immunization with plasmid DNA for the catalytic domain of TS elicited protective activity against *T. cruzi* infection in BALB/C mice (Costa et al., 1998). The IgG1 antibody class is the predominant one detected in the serum of immunized mice, being able to inhibit TS activity *in vitro*. Similarly, mice actively immunized and subsequently challenged with bloodstream trypomastigotes had a reduction in peak parasitemia and mortality. Although the study did indeed evaluated as occurs this protective response, recombinant TS evoked a significant type IV hypersensitivity response, a unique type of mediated immunity cells, and induced an intense proliferation in lymphoid cells derived from the lymph node and spleen of immunized mice (Costa et al., 1998).

The following year, a study compared the effects of immunogenicity from plasmids containing the catalytic domain gene of *T. cruzi* TS and recombinant and native TS. Both forms of immunization produced a peak humoral response after 3-4 doses, having no additive effect with the administration of a fifth dose (Pereira-Chioccola et al., 1999). Any immunization originated from the recombinant as native TS produced ten times more antibody titers that performed TS plasmid. Again, the two forms of immunization produced antibodies capable of inhibiting TS activity *in vitro*, requiring lower concentrations of antibodies from immunization with the protein (Pereira-Chioccola et al., 1999). Unlike Costa et al.’s results (Costa et al., 1998), naked DNA immunization produced mostly IgG2a class antibodies that did not protect mice from death after challenge with *T. cruzi*. On the other hand, immunization with the recombinant protein mainly produced IgG1 antibodies and 60% of the immunized mice survived the infection (Pereira-Chioccola et al., 1999). Thus, the authors speculate that immunization with recombinant TS with aluminum as weak adjuvant generated a Th2-type immune response, while naked DNA immunization generated a Th1-type response. It is noteworthy the difference in the survival of animals immunized and challenged with the Y strain of *T.cruzi* compared to the study by Costa et al. (1998). However, this change may be due to the murine model used in the studies. The murine A/Sn strain is more sensitive to Y strain infection than the BALB/C strain. The amount needed to kill most animals of the first lineage is less than 250 bloodstream trypomastigotes, while for the last strain, at least 1500 parasites are necessary (Costa et al., 1998; Pereira-Chioccola et al., 1999; Gamba et al., 2021). Later, another study confirmed this immunomodulatory duality in vaccine with DNA versus recombinant protein, demonstrating that the simultaneous administration of both types of immunization (DNA vaccination + recombinant protein) still induced a Th2 type response. However, when two doses of TS gene DNA vaccine were administered prior to vaccination with recombinant TS, the response reverted to the Th1 type (Vasconcelos et al., 2003).

Cells from the lymph node of BALB/C mice vaccinated with the TS gene were characterized as gamma-interferon (INF-γ)-producing Th1-type CD4 T cells, with highly cytotoxic activity *in vitro* and capable of inhibiting the development of *T. cruzi* in infected macrophages (Rodrigues et al., 1999). Surprisingly, the production of Th2-type cytokines such as IL-4 and IL-10 has been identified in some CD4 T cell clones. Additionally, CD8 T cells derived from the spleen of the immunized animals showed uniform response mediated by cytotoxic T lymphocytes (CTL), which also secrete INF-γ and induce lysis and DNA degradation of cells expressing TS (Rodrigues et al., 1999). Generally, vaccines that use naked DNA produce a Th1-type immunomodulatory response due to the presence of Cpg oligopeptides in bacterial plasmids (Klinman et al., 1997; Leclerc et al., 1997). Clearly, the TS gene induces predominantly Th1 type cells, but also induces Th2 type cells (Pereira-Chioccola et al., 1999; Rodrigues et al., 1999), being possible the coexistence of these two populations as verified in DNA vaccine models with other genes (Mor et al., 1995; Chow et al., 1998).

To unravel the immunodominant epitopes of this response, Martin and colleagues revealed that a single TS epitope was responsible for stimulating more than 30% of circulating INF-γ-producing CD8 T cells from C57BL/6 mice infected with Brazil strain *T. cruzi* (Martin et al., 2006). Factors that appear to contribute to immunodominance include abundance and expression kinetics, release of peptides by cellular proteases, peptide affinity for MHC and CD8 T cell repertoire. Interestingly, this immunodominance for a single epitope is extremely high, as the typical frequencies reported for the same assay range from 2-20% of CD8 T cells from mice infected with other diseases (Murali-Krishna et al., 1998). This ability of CD8 T cells to respond to a pool of few epitopes has also been observed in patients chronically infected with *T. cruzi* (Martin et al., 2006). In contrast, peptides encoded by genes from cruzipains, mucin-associated surface proteins, β-galactofuranosyl transferase and gp63 proteins showed a 4 to 20-fold lower frequency of stimulation of INF-γ-producing cells compared to TS epitopes, suggesting that specificity of epitopes of CD8 T responses in experimental *T. cruzi* infection is strongly dominant by peptides encoded by the TS family (Martin et al., 2006). However, Tzelepis et al. provide evidence that this could be explained by competition of T cells by APC, as mice infected simultaneously with two strains of parasites containing different immunodominant peptides generated maximal responses to both peptides (Tzelepis et al., 2008). The interpretation was that whether the peptides are presented by different APCs, the immunodominant pattern was disrupted. Thus, the immunodominance mechanism would be a mechanism used by the parasite to reduce the immune response and favor the progression to the chronic phase of the disease. Two years later, Rosemberg and colleagues induced simultaneous tolerance to two immunodominant *T. cruzi* epitopes in a resistant mouse strain. After infection, there was an increased susceptibility to infection, but the animals were still controlled and survived the infection (Rosenberg et al., 2010). The authors suggested that the response was mediated by CD8 T cells specific for subdominant epitopes that would replace the immunodominant ones. Dominguez et al. demonstrate that induction of response to subdominant antigens provides some degree of protection, although it does not produce an optimal response compared to immunodominant epitopes (Dominguez et al., 2011). Consequently, an artificial amplification of the immune response to subdominant antigens could be a strategy to improve the immunity induced by vaccination, favoring the host.

Despite the immunodominance capacity of TS peptides to stimulate CD8 CTL in mice infected with *T. cruzi* (Martin et al., 2006), vaccines with recombinant TS produce a predominantly Th2 response with activation of CD4 T cells, although they can protect immunized mice (Pereira-Chioccola et al., 1999). Parasitic diseases such as Leishmaniasis, Malaria and Chagas disease induce a large CD8 T response that is crucial for the resolution of the infection (Belkaid et al., 2002; Morrot and Zavala, 2004). Likewise, a vaccine model that induces a strong activation of CD8 CTL appears to contribute to a satisfactory protective response (Dumonteil, 2009). Generally, immunization with protein subunits does not stimulate CD8 CTL, as exogenous antigens are absorbed by endocytosis by antigen-presenting cells (APC), generating epitopes that are processed and presented exclusively *via* MHC class II, recognized only by CD4 T cells. However, some APCs are able to perform an alternative mechanism of cross-presentation of exogenous antigens *via* MHC class I, priming CD8 CTL in a process known as cross-priming (Rock and Shen, 2005). It is suggested that parasitic infections, such as Chagas disease, the induction of a TCD8 response comes from immunodominant peptides that may be favored in cross-priming in relation to subdominant peptides (Dominguez et al., 2011). This would explain why CD8 T cells respond to only a reduced pool of TS peptides.

Hoft and collaborators synthesized a Cpg mixed TS vaccine (TS/Cpg) administered intranasal or intramuscularly that induced systemic immunity in BALB/C mice challenged with the Tulahuen strain of *T. cruzi*, producing TS-specific IgG antibodies and reducing the mortality of infected animals. Immunization with intranasal TS/CpG mobilized activation of CD4 Th1 cells, as well as CD8 CTL with potential cross-presentation of antigens by B cells (Hoft et al., 2007). Furthermore, previous immunization with TS/Cpg induced the production of IgA detected in fecal extracts and significantly reduced the number of viable parasites in draining gastric lymph nodes and recoverable molecular equivalents of parasite from the gastric epithelium after oral infection, demonstrating in addition to systemic protective immunity also mucosal immunity (Hoft et al., 2007). Intranasal administration of vaccines is even more advantageous since DNA vaccines are degraded and/or are not well absorbed *via* the mucosa.

For over 20 years, it has been discovered that TS gene vaccination induces Th1 immunity in mice and other laboratory animals (Dumonteil et al., 2012; Luna and Campos, 2020). However, these DNA vaccines have not advanced to the clinical trial phases, possibly because they are not as efficient or safe for testing in humans (Schalk et al., 2006; Hobernik and Bros, 2018). On the other hand, immunization with recombinant protein subunits associated with adjuvants is less disputed regarding safety, being better accepted for clinical trials by regulatory agencies (Dumonteil et al., 2012). Aware of these facts, Fontanella and collaborators designed a vaccine candidate with a mutant TS (mTS - enzymatically deficient containing the catalytic domain and without the SAPA repeats), which showed a great protective response to infection by the Tulahuen strain in BALB/C mice in relation to those not immunized or immunized with recombinant TS (Fontanella et al., 2008). For the first time, an immunization protected 100% of the animals and prevented the development of tissue damage, especially in the myocardium. Knowing the role of TS in the pathogenesis of Chagas disease, the design of a mutant candidate enabled a more appropriate protective response, since animals immunized with recombinant TS and challenged still exhibited tissue inflammation in skeletal and cardiac muscle, moderate splenomegaly and changes in hematocrit (Fontanella et al., 2008). Anti-SAPA antibodies were not found in immunized animals; they are not protective and seem to be associated with tissue damage and humoral response delay (Fontanella et al., 2008). Although Freund’s complete adjuvant (FCA) used in the work has an excellent performance, ensuring a mixed humoral and cellular response that is hardly surpassed by other adjuvants, FCA is not recommended for human use due to the injuries associated with its administration. Local necrosis and granulomatous inflammatory response are characteristics from FCA injection site (Stills, 2005).

With that in mind, Bontempi and colleagues used mTS with a new adjuvant called ISCOMATRIX (IMX), composed of saponin, cholesterol and phospholipids that combined form 40-50 nm like-cage structures. IMX was chosen due to the antigen trafficking more efficient and persistent to draining lymph nodes (up to seven days after injection). Moreover, it promotes epitope processing and addressing both *via* MHC class II, presenting to CD4 T cells and inducing a B cell response; and *via* MHC class I, facilitating the cross-priming of CD8 T cells (Baz Morelli et al., 2012). Thus, immunization with mTS-IMX equaled the effects with mTS-FCA in terms of TS-specific antibody production, avidity, type IV hypersensitivity response, Th1 profile, and reduction in mortality and tissue damage (Bontempi et al., 2015). Again, there was 100% survival of mice immunized and challenged with *T. cruzi*, even with ten times more inoculated bloodstream trypomastigotes than in the work by Fontanela et al. (Fontanella et al., 2008). Additionally, splenocytes from immunized animals showed a production of INF-γ five times greater than infected mice, in addition to the production of IL-10 that could be responsible for halting an exacerbated inflammatory response (Bontempi et al., 2015). Subsequently, immunization with mTS-IMX proved to be more efficient in inducing humoral and cellular responses when compared to other immunogenic proteins such as flagellar repetitive protein (which contains tandem repeats), tryparedoxin peroxidase (involved in the metabolic pathway) and cruzipain (involved in parasite invasion) using the same adjuvant (Bontempi et al., 2017).

Based on background of TS-based vaccines, Prochetto and collaborators engineered a new peptide of only 290 amino acids, missing the SAPA domain and the Nh2 terminal region, making it inactive (Prochetto et al., 2017). The reduced size of the TS fragment (TSf) would facilitate the development of vaccines, considering the expression of recombinant proteins in bacteria is improved when the size of the DNA sequences is less than 1000 base pairs (Makino et al., 2011). TSf showed 90% identity with the mTS sequence, derived from random mutagenesis in yeast, which showed a robust humoral and cellular response with IMX adjuvant, protecting 100% of the infected animals (Bontempi et al., 2015). TSf also showed 92% identity against TS sequences indexed in *GenBank* from different strains of *T. cruzi* (Prochetto et al., 2017). In addition, TSf was formulated with a new adjuvant called ISPA, composed of 70 nm-liposomes with a cage-like structure from phosphatidylcholine, cholesterol, sterylamine, tocopherol and saponin (Bertona et al., 2017). TSf-ISPA showed similar results to immunization with mTS-IMX promoting stimulation of several Th1 profile components. Interestingly, TSf-ISPA immunized mice challenged with *T. cruzi* showed an increase of regulatory T splenocytes (Treg, CD4+Foxp3+) compared to non-immunized and challenged mice, although there was no change in the percentage of the population (Prochetto et al., 2017). Treg cells constitute about 3-5% of the peripheral T cell population and have an important anti-inflammatory activity for the regulation of immune homeostasis (Richards et al., 2015). The authors believe that this increase has a beneficial effect on the survival of immunized animals and, possibly, in the chronic phase of Chagas disease, taking into account that asymptomatic patients have a greater number of Treg cells than symptomatic patients. On the other hand, immunization reduced the percentage and absolute number of myeloid-derived suppressor cells (MDSC), heterogeneous population of immature innate cells composed of monocytes, granulocytes and dendritic cells expressing CD11b and GR-1 in mice, able to potently suppress pro-inflammatory immune responses (Yaseen et al., 2021). In the proposed model by authors, the formulation with TSf-ISPA generates a better control of the inflammatory response induced by components of the Th1 response, increasing protective FoxP3 Treg cells and reducing MDSC-induced generalized immunosuppression (Prochetto et al., 2017).

Some infection studies have shown an improvement in the vaccine response associated with a reduction in MDSC (Sui et al., 2014; Prochetto et al., 2017; Dorhoi et al., 2020). Recently, Gamba and collaborators decided to evaluate the role of MDSC cell depletion by 5-fluoracil in the vaccine model with TSf-ISPA. The depletion of MDSC cells in BALB/C, 15 days after infection with *T. cruzi*, promoted an increase in parasitemia and 100% of the animals died by day 21, even with a lower infective dose of the Tulahuen strain (900 bloodstream trypomastigotes). This result is possibly due to an exacerbated pro-inflammatory response by CD8 T cells. In contrast, 60-100% of mice survived the challenge when they were previously immunized with TSf-ISPA, even with an almost complete reduction of the granulocytic population (G-MDSC) and partial reduction of the monocytic population (M-MDSC). These animals showed an increase in CD8 T cells, Treg cells and maturation of CD8 dendritic cells greater than mice immunized only and challenged with parasites, suggesting that modulation of MDSC cells may have beneficial effects on immunization against *T. cruzi*. Noteworthy, when MDSC depletion was early in immunized animals (day 5 post-infection or pre-immunization), there was a reduction in parasitemia and an increase in the survival of animals than late depletion (day 15 post-infection), even at lethal doses of *T. cruzi* (1500 bloodstream trypomastigotes) (Gamba et al., 2021) As a result, the possibility of modulating MDSC could be an interesting tool for the design of vaccines and adjuvants in TS-based vaccines.

The difficulty in producing vaccines when talking about Chagas disease is due to many factors. One of them is the great genetic and biochemical variability of the different strains of *T. cruzi* (Zingales, 2018). Since 2009, *T. cruzi* strains have been classified into six discrete typing units (DTU): I and II would be ancestral strains; III and IV, homozygous hybrids of ancestral strains; and V and VI, heterozygous hybrids of II and III (Zingales et al., 2009). TcBat is a new strain discovered in bats and has been considered as DTU VII (Lima et al., 2015). Breniere and collaborators provided an inventory with 6343 DTU analyzed according to geographic region and host origin. Although *T. cruzi* is considered a diploid organism, it presents variations in size and number of chromosomes between strains and within the same clone, demonstrating great genomic plasticity (Reis-Cunha et al., 2018). The TS family genes are part of the non-systemic disruptive compartment and are considered recent and still evolving. Possibly, its location close to telomeric and sub-telomeric regions favors genetic variability of this family due to the occurrence of recombination events (Chiurillo et al., 2016; Ramirez, 2019). This could generate a pool of different phenotypes within the same population and increase adaptability to different selective pressures, such as the evasion capacity of the immune system (Herreros-Cabello et al., 2020). In addition, the parasite has different evolutionary stages in host cells, expressing different patterns of surface glycoproteins that make it difficult for the host antibodies to recognize antigens. Additionally, the parasite has a great capacity to subvert pro-inflammatory immune responses using dual mechanisms to allow its permanence in the host (Cardoso et al., 2015; Da Fonseca et al., 2019). Therefore, these factors must be taken into account when developing TS-based vaccines.

**Figure 2:**
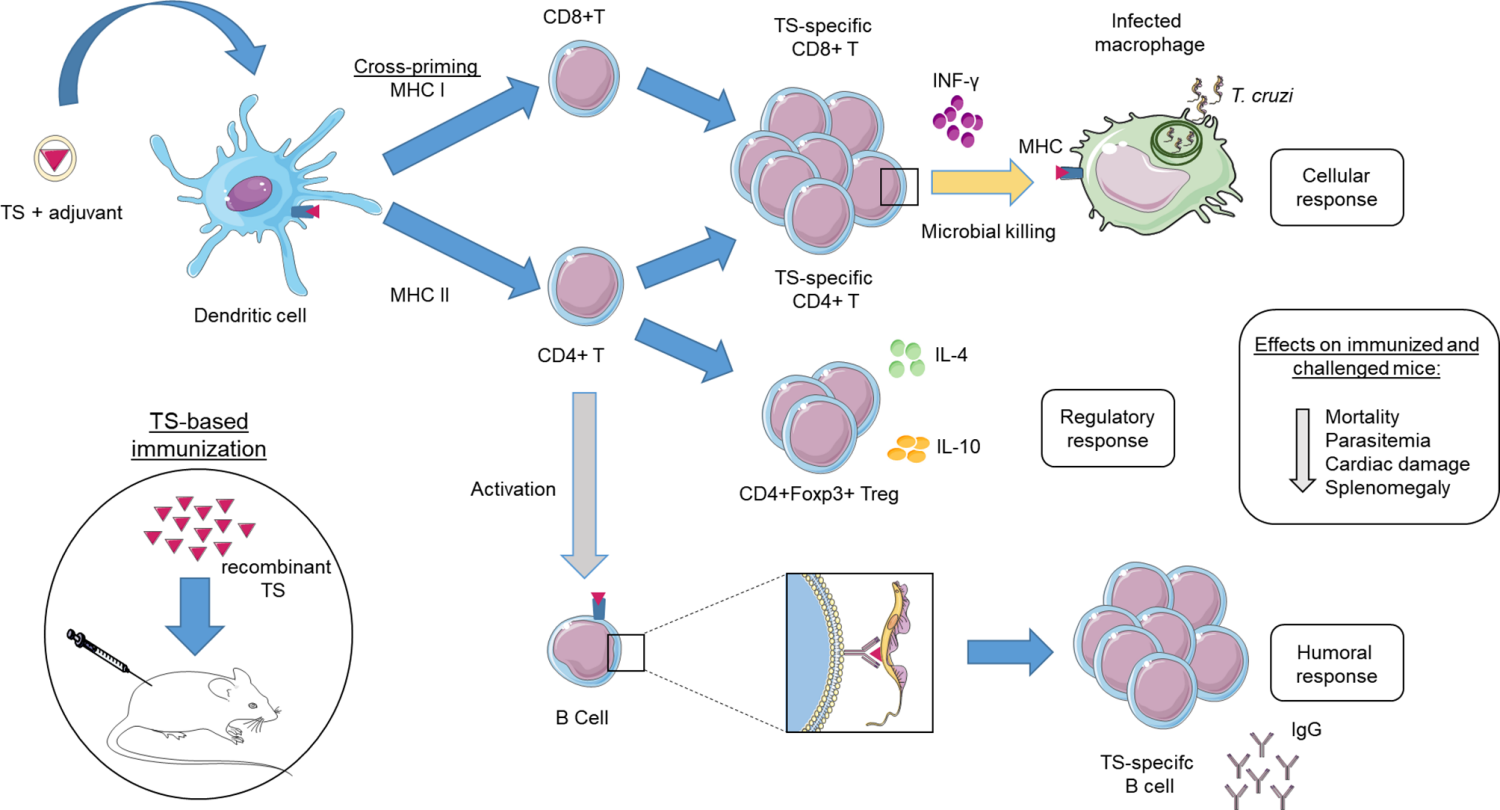
Immune response model for *trans*-sialidase (TS)-based vaccines. In the immunization of mice with vaccines based on recombinant TS, exogenous antigens are usually processed and presented by antigen presenting cells (APC) *via* MHC class II, recognized by CD4 T cells. This pathway will stimulate B cells to produce and secrete TS-specific antibodies (Humoral response). Certain types of APC such as dendritic cells can process and present exogenous antigens *via* MHC class I, recognized by CD8 T cells, a process known as cross-priming. Both TS-specific CD8 and CD4 T cells can differentiate into cells with cytotoxic activity capable of killing *Trypanosoma cruzi*-infected cells as macrophage (Effector response). In addition, a group of specific-TS CD4 T cells (Foxp3) known as regulatory T cells are responsible for controlling the pro-inflammatory response in the host (Regulatory response). The immunization of animals with vaccines based on TS has reduced parasitemia, tissue damage and consequently the mortality rate.

The success of a recombinant TS vaccine for *T. cruzi* depends on its ability to produce an epitope-specific humoral response that experiences little variability between strains. As it is an exogenous protein, its antigens will be processed by APC and presented *via* MHC class II, recognized by CD4 T cells, which will induce stimulation of antibody-producing B cells. Certain types of APC can perform cross-presentation of antigens *via* MHC class I, recognized by CD8 T cells (**Figure 2**). This cross-presentation is enhanced with the use of a new generation of adjuvants as IMX and ISPA, being essential for a long-lasting protective response in subunit vaccine. Furthermore, these new adjuvants showed rapid and persistent antigenic delivery to APC as well as a robust Th1 response similar to FCA without causing adverse effects on immunization, making them safer for testing in humans. Moreover, it is normal for antibody titers to decrease over time in immunized and unstimulated individuals, reinforcing the contribution that cellular response can make at the onset of infection. However, an exacerbated inflammatory response can induce intense tissue inflammation, producing unwanted clinical manifestations. Thus, vaccine candidates need to have a brake on the pro-inflammatory response. This function seems to be performed by the Treg cells in a murine model, which can be stimulated by reducing the MDSC population. As reported in the text, TS-based candidates have achieved high survival rates in animal model and reduced parasitemia. The increase in survival is essential but reducing the number of parasites is also an important parameter because it is associated with reduction of clinical symptoms in the chronic phase.

The development of recombinant vaccines against Chagas disease, in addition to focusing on sequences that present great immunogenicity, should overcome the problem of the great genetic variability of the strains. An inventory with 6343 strains divided into the six existing DTUs according to their geographic location and host origin showed that strains belonging to DTU I are more frequent (found in more than 60% of the analyzed samples) and widely distributed geographically (Breniere et al., 2016). Moreover, they cause the clinical manifestation of cardiac disease, with high morbidity, and are also found in patients infected by the oral route (Zingales et al., 2012; Zingales, 2018). For this reason, vaccines may initially focus on DTU I strains due to their epidemiological impact. Regarding the TS multigene family, group I presents the characteristic sequences of the family (VTVxNVxLYNR) and other conserved sequences such as Asp-box (SxDxGxTW) and SAPA that must be taken into account in the design of the recombinant vaccine (Berna et al., 2018). It is noteworthy that similarities were found between the sequence of group I members of the *T. cruzi* TS with other species of the genus (Herreros-Cabello et al., 2020), suggesting the possibility of highly conserved sequences. In this way, we hope to have listed the strengths and weaknesses of TS-based vaccines and we believe that this protein due to its immunogenicity and conserved sequences still constitutes a great target to produce of a vaccine based on recombinant proteins that are cost effective on large scales and are safer when compared to other formulations.

## Author Contributions

KC and LM searched the bibliographic materials, reviewed the existing literature, and wrote the article. JR and MS reviewed the literature. LFL, JP, and LMP supervised the work. All authors contributed to the article and approved the submitted version.

## Funding

The study was supported by Fundação Carlos Chagas Filho de Amparo à Pesquisa do Estado do Rio de Janeiro and Conselho Nacional de Desenvolvimento Científico e Tecnológico.

## Conflict of Interest

The authors declare that the research was conducted in the absence of any commercial or financial relationships that could be construed as a potential conflict of interest.

## Publisher’s Note

All claims expressed in this article are solely those of the authors and do not necessarily represent those of their affiliated organizations, or those of the publisher, the editors and the reviewers. Any product that may be evaluated in this article, or claim that may be made by its manufacturer, is not guaranteed or endorsed by the publisher.

## Abbreviations

APC, antigen-presenting cells; CTL, cytotoxic T lymphocytes; G-MDSC, granulocytic myeloid-derived suppressor cells; Ig, immunoglobulin; IMX, ISCOMATRIX; INF-γ, gamma interferon; iTS, inactive *trans*-sialidase; Mab, monoclonal antibodies; MDSC, myeloid-derived suppressor cells; M-MDSC, monocytic myeloid-derived suppressor cells**;** mTS, mutant trans-sialidase; SAPA, shed acute phase antigen; SCID, severe combined immunodeficiency**;** Sia, sialic acid; Th, T helper; Treg, Foxp3+ CD4+ T cells; TS, *trans*-sialidase; TSf, trans-sialidase fragment.
